# Reagent-free sonochemical synthesis of iron oxide nanoparticles from iron powder and water promoted by acoustic cavitation^☆^

**DOI:** 10.1016/j.ultsonch.2026.108028

**Published:** 2026-08-25

**Authors:** Madoka Yoshikawa, Hirotsugu Takizawa, Yamato Hayashi

**Affiliations:** Graduate School of Engineering, Department of Applied Chemistry, Tohoku University, 6-6 Aoba, Aramaki, Aobaku, Sendai 980-8579, Japan

**Keywords:** Magnetite, Maghemite, Nanoparticles, Iron powder, Ultrasound

## Abstract

This study demonstrates a distinct reagent-free sonochemical route in which acoustic cavitation directly converts elemental Fe powder into spinel-type iron oxide nanoparticles in water at near-ambient temperatures, without soluble Fe precursors, precipitating agents, or other added reagents. At 43 kHz and 40 °C for 24 h, the obtained particles exhibited a mean diameter of 32 nm and a magnetization of 85.6 emu/g at the maximum applied field. Under otherwise identical conditions at 60 °C, particles with a mean diameter of 33 nm and a magnetization of 76.9 emu/g were obtained. The difference in magnetization may reflect changes in the Fe^2+^/Fe^3+^ balance and/or nanoscale magnetic effects, although the phase composition could not be determined quantitatively from the present measurements. Based on the observed morphologies and phase evolution, nanoparticle formation is proposed to involve both homogeneous nucleation from dissolved Fe species and heterogeneous oxidation on the bulk Fe surface. In contrast, mechanical stirring induced Fe oxidation but produced submicrometer-sized particles attached to the Fe surface. Acoustic cavitation may disrupt the surface oxide layer, expose fresh Fe interfaces, and thereby promote the formation and detachment of fine particles. These findings demonstrate acoustic-cavitation-assisted direct metal-to-oxide conversion as a synthesis pathway distinct from conventional iron-salt-based precipitation, while avoiding soluble Fe precursors and precipitating agents.

## Graphical abstract

**Figure:**
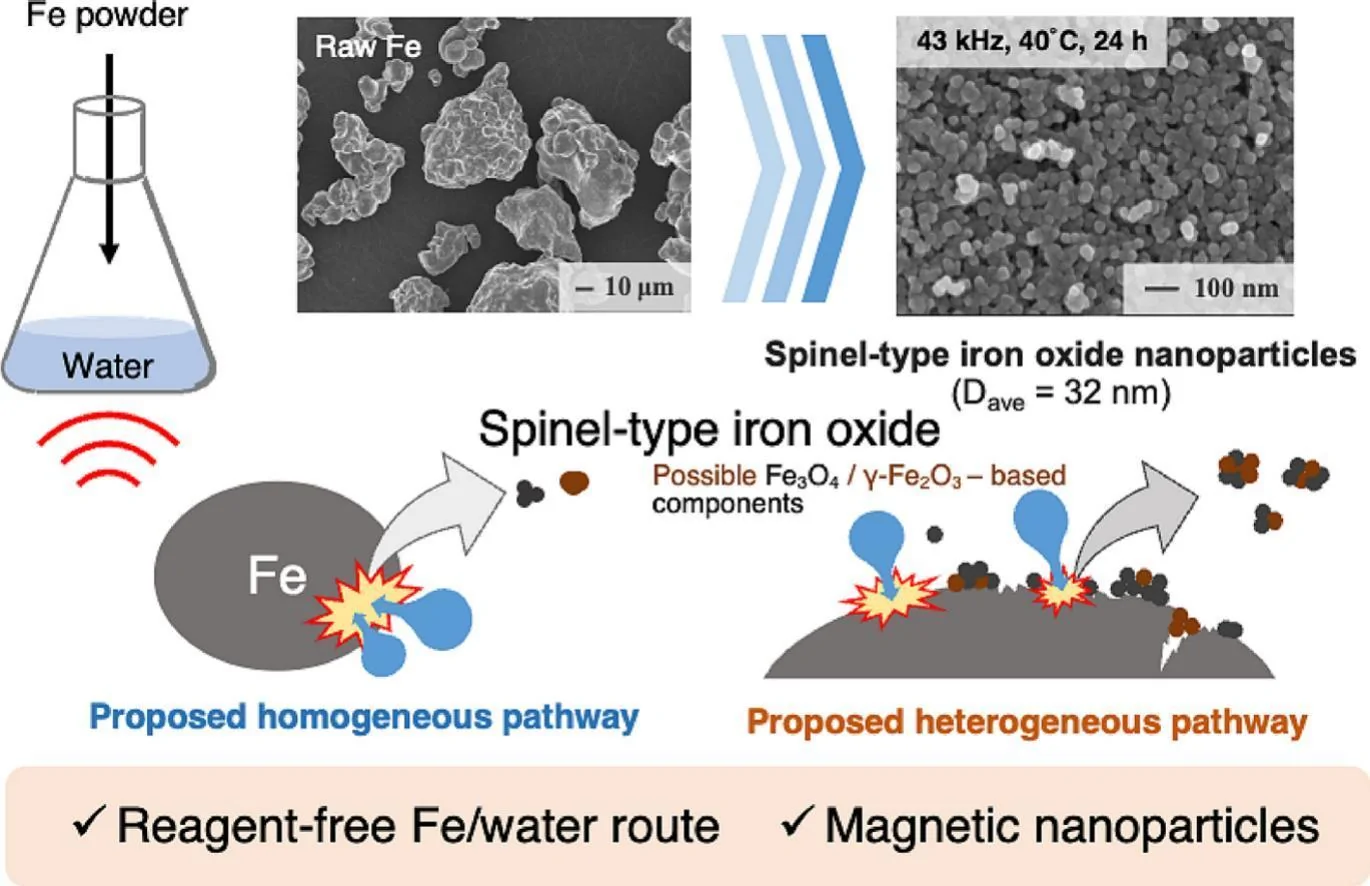

## 1 Introduction

Maghemite (γ-Fe_2_O_3_) and magnetite (Fe_3_O_4_) are among the most common naturally occurring iron oxides [1], and both exhibit soft ferrimagnetic behavior at room temperature [2]. Fe_3_O_4_ is a mixed-valence oxide containing both Fe(II) and Fe(III), and possesses a cubic inverse spinel structure. γ-Fe_2_O_3_ has a similar inverse spinel structure to Fe_3_O_4_; all iron ions are present in the fully oxidized Fe^3+^ state [3]. Iron oxide nanoparticles are known to exhibit high stability in air and possess various properties, including biocompatibility [4], adsorption [5], and superparamagnetism [6]. Iron oxide nanoparticles have been applied in a wide range of fields, including ferrofluids [7], magnetic resonance imaging (MRI) [8], magnetic hyperthermia [9], and drug delivery systems, depending on their size and morphology. The major synthesis methods for iron oxide nanoparticles are summarized in Table 1. As summarized in Table 1, previously reported synthesis methods produce iron oxide nanoparticles with particle sizes ranging from approximately 5 to 331 nm and generally require reaction times ranging from approximately 1 to 24 h, depending on the synthesis route. The present sonochemical method produced spinel-type iron oxide nanoparticles with a mean particle size of 32 nm after 24 h at 40 °C. Although the reaction time of the present method is relatively long, it differs from many conventional precipitation-based methods in that Fe powder and water are used without soluble Fe salts or added precipitating agents. Therefore, the principal advantage of the present method is the simplification of chemical inputs and downstream purification rather than a demonstrated reduction in reaction time or energy consumption.

**Table 1:** Comparison of synthesis conditions and particle sizes of iron oxide nanoparticles prepared by previously reported methods and the present sonochemical method.

**Method**	**Products**	**Precursors**	**Temperature / ℃**	**Reaction time / h**	**Particle size / nm**	**Ref.**
Solvothermal	Fe_3_O_4_	FeCl_3_·6H_2_O	200	8	49.3–85.7	[10]
*In situ* precipitation	Fe_3_O_4_	FeCl_2_·4H_2_O, FeCl_3_·6H_2_O	60	1.5	9–15	[11]
Hydrothermal	Fe_3_O_4_	FeCl_3_·6H_2_O	120	4	23.46	[12]
Green	Fe_3_O_4_	FeSO_4_·7H_2_O	90	1	23.60	[13]
Electrochemical	Fe_3_O_4_	FeSO_4_·7H_2_O	Ambient	6	19–23	[14]
Co-precipitation	Fe_3_O_4_	FeCl_2_·4H_2_O, FeCl_3_·6H_2_O	25–80	1	52–331	[15]
Co-precipitation	Fe_3_O_4_	FeCl_2_·4H_2_O, FeCl_3_·6H_2_O	−	−	10–12	[16]
Sol-gel	Fe_3_O_4_	Fe(NO_3_)_3_·9H_2_O	85, 130–170	4–10	4.71–5.91	[17]
High-energy ball milling	Fe_3_O_4_, γ-Fe_2_O_3_	α-Fe_2_O_3_	−	24	15	[18]
Co-precipitation	γ-Fe_2_O_3_	FeSO_4_, FeCl_3_	Ambient, 300–500	−	20	[19]
Solid-phase reduction–oxidation reaction	γ-Fe_2_O_3_	FeSO_4_, FeS_2_	550, 350	1.5	35	[20]
Co-precipitation	Fe_3_O_4_, γ-Fe_2_O_3_	FeCl_2_·4H_2_O, FeCl_3_·6H_2_O	70, 100	1, 2	9.31, 16.30	[21]
Flame spray pyrolysis	Fe_3_O_4_, α-Fe_2_O_3_, γ-Fe_2_O_3_	Fe(NO_3_)_3_	1910	−	8.7	[22]
Sol-gel	γ-Fe_2_O_3_	Fe(NO_3_)_3_·9H_2_O	50, 150	14	7.2	[23]
Sonochemical*	Spinel-type iron oxide	Fe powder, H_2_O	40	24	32	Present study

*The values for the present study correspond to ultrasonic irradiation at 43 kHz and 40 °C for 24 h.

Iron oxide nanoparticles are commonly synthesized by co-precipitation, hydrothermal, solvothermal, sol-gel, electrochemical, and flame-based processes. These methods enable control over particle size, morphology, and phase composition; many employ iron chlorides, sulfates, nitrates, or organometallic compounds as precursors. Precursor-derived ionic species and other byproducts must be removed through washing, separation, and wastewater treatment. Hydrothermal, solvothermal, and thermal decomposition processes often require elevated temperatures and/or pressures, thereby increasing energy consumption and process complexity. Reducing the use of salt precursors and simplifying downstream purification are therefore important challenges in the sustainable synthesis of iron oxide nanoparticles.

Ultrasound has been widely employed to enhance mixing, nucleation, and mass transfer during the co-precipitation of iron salts. In most reported sonochemical processes, ultrasound acts primarily as an auxiliary energy source in conventional precursor systems, and metal salts, pH-adjusting agents, or other reagents remain necessary. The direct sonochemical conversion of metallic Fe into spinel-type iron oxide nanoparticles using only water under near-ambient conditions remains insufficiently investigated. The contribution of acoustic cavitation to Fe-surface renewal, oxidation, particle detachment, and nanoscale particle formation has not been clearly established. Accordingly, a key unresolved question is whether acoustic cavitation can directly drive the solid-liquid transformation of elemental Fe into spinel-type iron oxide nanoparticles in water and which cavitation-induced interfacial processes may contribute to this conversion. This distinction between conventional iron-salt-based precipitation and the present direct metal-to-oxide route is summarized in Table S3.

This study aimed to develop a reagent-free sonochemical route for synthesizing spinel-type iron oxide nanoparticles directly from Fe powder and water at near-ambient temperatures and atmospheric pressure. The effects of irradiation time, ultrasonic frequency, and reaction temperature were investigated, and the role of acoustic cavitation was examined by comparison with mechanical stirring.

The Fe powder has the potential to serve as a raw material derived from scrap iron. Scrap is a resource consisting of discarded iron products and Fe waste generated during the processing of steel and other Fe-based materials. Scrap Fe generally consists of fine particles and is readily oxidized owing to its high reactivity [24]. This poses a major challenge to many existing recycling processes. By contrast, as this study aimed to synthesize metal oxide particles from fine metal particles, this limitation was advantageous.

Nanoparticle synthesis using ultrasound enables reactions to proceed under milder conditions than those used in conventional methods. Numerous microscopic bubbles are generated when ultrasound is applied to a liquid. These bubbles grow while repeatedly expanding and contracting in response to the pressure fluctuations induced by ultrasonic waves [25]. The bubbles eventually collapse, generating localized high-temperature and high-pressure reaction zones known as hotspots. Representative values reported for cavitation hotspots can exceed approximately 5000 K and 1000 atm [26]; these localized conditions were not measured directly in the present reactor. In addition to these chemical effects, bubble collapse generates the physical effects of microjets and shock waves [27], including agitation [28], erosion [29], and damage of solid surfaces [30]. Although these extreme conditions are generated by cavitation, they are confined to highly localized regions, allowing the reaction system to remain under ambient temperature [31].

Several studies have reported the synthesis of iron oxide nanoparticles using ultrasound. In many of these studies, ultrasound is primarily employed to enhance mixing [32] and promote reactions in co-precipitation [33] or reverse co-precipitation [35]. Sono-assisted preparation of Fe_3_O_4_ nanoparticles for catalytic removal of organic pollutants using H_2_O_2_ has also been reported [34]. As the precursor systems and processing conditions are largely comparable to those listed in Table 1, environmentally benign synthesis methods that avoid the generation of byproducts and wastewater are still under development. These previous studies demonstrate the usefulness of ultrasound in precursor-based iron oxide synthesis; however, they do not establish a direct cavitation-driven transformation of elemental Fe into nanoscale iron oxide in water. The novelty of the present study therefore lies in demonstrating this direct solid-liquid metal-to-oxide transformation and in clarifying the role of acoustic cavitation by comparison with mechanical stirring.

This research group has previously reported the synthesis of various metal and metal oxide particles via ultrasonic irradiation in liquid-phase systems, including silver nanoparticles [36], copper nanoparticles [37], nickel microparticles [38], Au-Ag alloy particles [39], γ-gallium oxide (γ-Ga_2_O_3_) nanoparticles [29], and boehmite (γ-AlOOH) nanopowders [40]. Notably, γ-Ga_2_O_3_ nanoparticles and γ-AlOOH nanopowders were synthesized directly from metallic Ga and Al, via ultrasonic irradiation at room temperature. Metallic Fe is inherently unstable in the presence of water and oxygen and readily undergoes oxidation. This suggests that iron oxide particles can be synthesized via ultrasonic irradiation. In this study, the sonochemical synthesis of iron oxide nanoparticles was investigated by ultrasonic irradiation of Fe powder in water. The effects of ultrasonic parameters, such as irradiation time and frequency, were examined to develop a process that enables synthesis at temperatures close to room temperature.

## 2 Experimental methods

The materials, synthesis procedure, control experiment, and characterization methods used in this study are described below.

### 2.1 Chemicals

Fe powder (98%, Kobe Steel Ltd.) was used as the starting material. Deionized water was used as the solvent. All chemicals were used as received without further purification.

### 2.2 Synthesis of iron oxide nanoparticles

Reaction temperatures of 40 and 60 °C were examined as the main experimental conditions. In addition, an experiment at 30 °C was conducted at 43 kHz for 24 h to extend the temperature range for evaluating the effect of reaction temperature. These temperatures were selected to examine the influence of reaction temperature on nanoparticle formation, Fe oxidation, and the structural characteristics of the resulting spinel phase under relatively mild aqueous conditions. Before the addition of Fe powder, the water was pre-sonicated for 30 min to reduce variations in the initial dissolved-gas content and improve the reproducibility of the cavitation conditions. After the addition of 1.0 g of Fe powder, ultrasonic irradiation was carried out at 23 or 43 kHz and 150 W for 1–72 h. The two frequencies were compared to evaluate the influence of ultrasonic frequency under the same nominal power condition, whereas the irradiation-time range was selected to follow the temporal evolution of Fe oxidation, nanoparticle formation, and particle coarsening. For samples irradiated for 24 h or less, the products were separated into the supernatant and residual solid fractions for separate analysis. For the 72 h sample, the supernatant fraction was also collected for SEM observation and particle-size analysis, whereas the entire recovered product was used for XRD analysis. The collected dispersions were vacuum-dried to obtain powdered samples. A Sonoreactor (Honda Electronics Co., Ltd.) was used for ultrasonic irradiation. The acoustic power delivered to the liquid was evaluated calorimetrically using 100 mL of water. The temperature increase after the ultrasound was switched on was fitted by linear regression, and the calorimetric power was calculated using *P_cal_* = *mc_p_* (d*T*/d*t*), where *m* and *c_p_* are the mass and specific heat capacity of water, respectively. Heating rates of 0.0299 and 0.0313 K s^−1^ were obtained at 23 and 43 kHz, respectively, corresponding to calorimetric powers of 12.5 and 13.1 W. The water bath temperature was maintained using a recirculating chiller (CTP-3000; Tokyo Rikakikai Co., Ltd.). A schematic of the ultrasonic operating system and the calorimetric power determined at each frequency are shown in Fig. S1 and Fig. S2.

To distinguish the specific effects of acoustic cavitation from those of macroscopic liquid mixing, a control experiment was conducted using mechanical stirring instead of ultrasonic irradiation. A flask containing degassed water and Fe powder was placed in a thermostatic water bath maintained at 40 °C and stirred at 300 rpm for 72 h with a mechanical stirrer (LM200; Yamato Scientific Co., Ltd.).

### 2.3 Characterization

The crystal phases of the samples were analyzed using X-ray diffraction (XRD; Bruker AXS). Cu Kα radiation (λ = 0.154184 nm) was used, and diffraction patterns were collected over a 2θ range of 10–80°. The XRD patterns used for Rietveld refinement were collected over a 2θ range of 10–110°. The lattice parameters and crystalline phase fractions were refined by Rietveld refinement using a modified split pseudo-Voigt profile function (Fig. S3). The morphologies of the samples were evaluated using field-emission scanning electron microscopy (FE-SEM; JSM-7610F, JEOL Ltd.). The magnetic properties of the products were characterized by measuring their magnetization curves at 300 K up to 20 kOe using a superconducting quantum interference device (SQUID; MPMS-5S, Quantum Design). Particle-size distributions were determined from 515 to 732 particles measured in at least two independent SEM images for each representative sample using ImageJ. The particle diameter was defined as the equivalent circular diameter calculated from the projected particle area, and the results are presented as histograms in Figs. S4–S6.

## 3 Results and discussion

### 3.1 Sample characterization

**Fig. 1:**
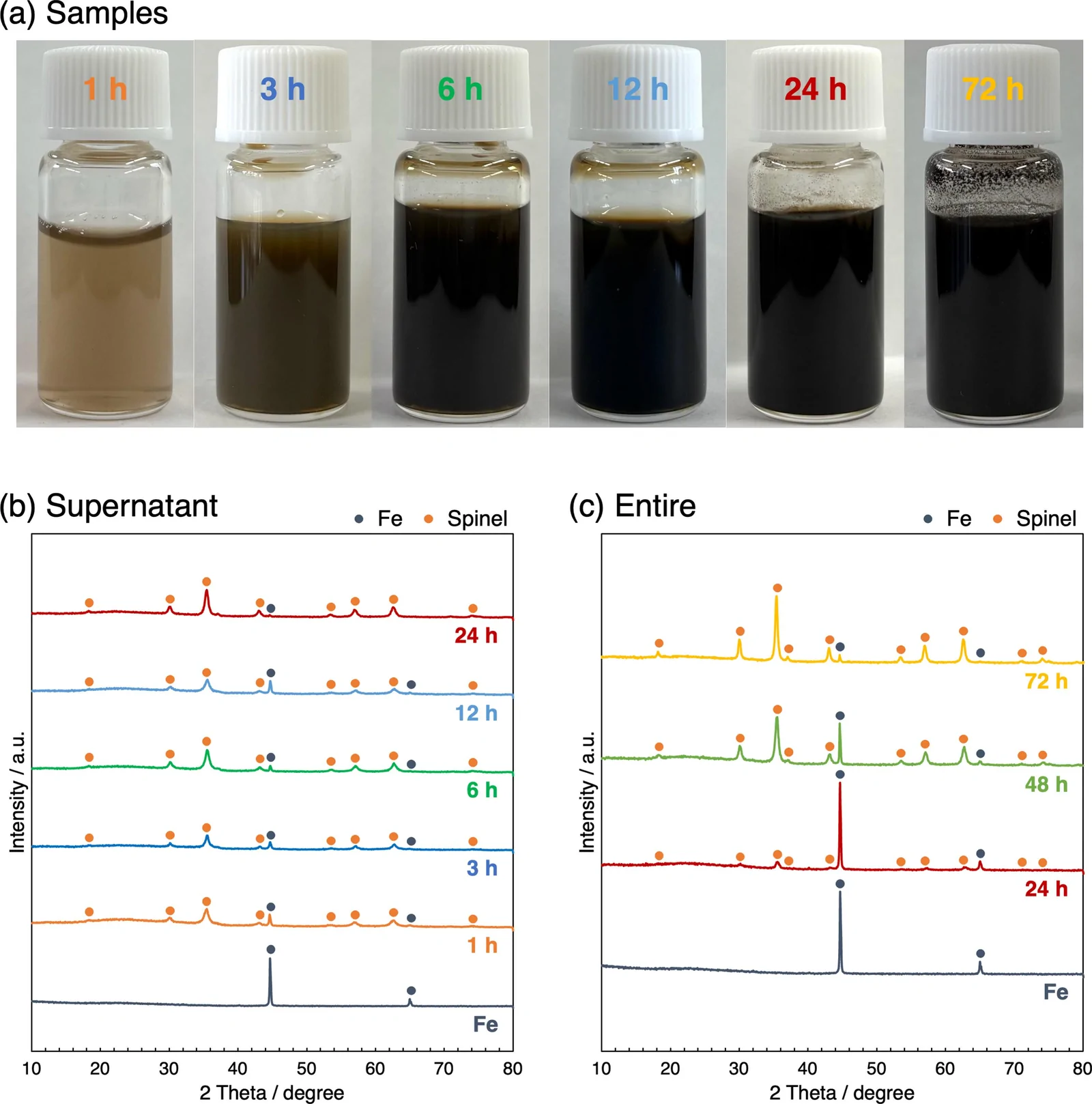
Photographs and X-ray diffraction (XRD) patterns of products obtained by ultrasonic irradiation of Fe powder in water at 23 kHz and 40 °C for different irradiation times: (a) photographs of the reaction suspensions, (b) XRD patterns of powders recovered from the supernatant after 1–24 h of irradiation, and (c) XRD patterns of the entire recovered products after 24–72 h of irradiation.

Fig. 1**(a)** shows photographs of the samples synthesized by ultrasonic irradiation of Fe powder in water at 40 °C (23 kHz). The XRD patterns of the supernatant-derived powders obtained after 1–24 h of sonication are presented in Fig. 1**(b)**, while those of the entire products synthesized after 24–72 h are shown in Fig. 1**(c)**. The color of the suspension darkened progressively with increasing reaction time. The XRD patterns exhibited diffraction peaks corresponding to a spinel phase attributable to Fe_3_O_4_, γ-Fe_2_O_3_, or both, although no peaks associated with other oxides or hydroxides were detected. These results indicate that iron oxide can be synthesized simply by ultrasonic irradiation of Fe powder in water. The intensity of the iron oxide peaks increased with irradiation time, suggesting progressive formation of the crystalline spinel oxide phase.

**Fig. 2:**
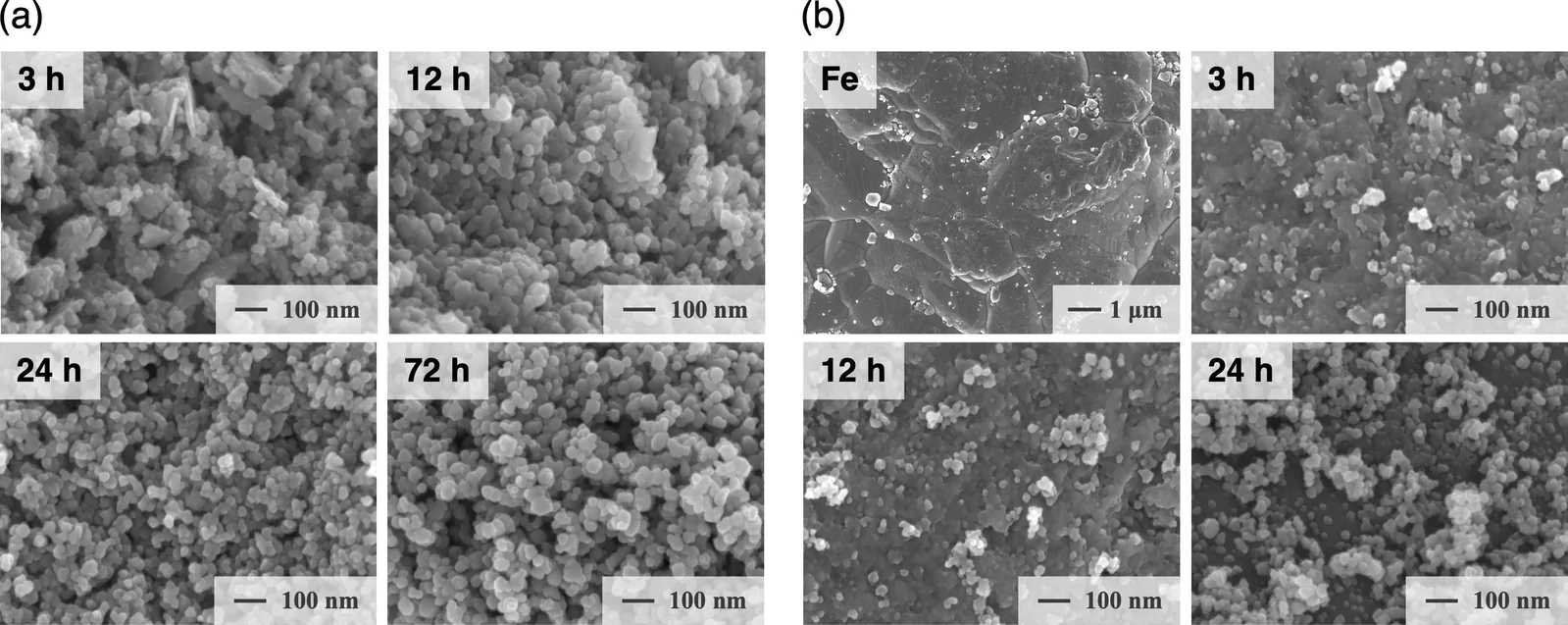
SEM images of samples obtained by ultrasonic irradiation of Fe powder in water at 23 kHz and 40 °C: (a) powders recovered from the supernatant after 3, 12, 24, and 72 h of irradiation and (b) starting Fe powder and residual solids recovered after 3, 12, and 24 h of irradiation.

Fig. 2 shows scanning electron microscopy (SEM) images of samples synthesized by ultrasonic irradiation of Fe powder in water at 40 °C. For morphological comparison, Fig. 2**(a)** shows the powders recovered from the supernatant after sonication for 3, 12, 24, and 72 h, whereas Fig. 2**(b)** shows the starting Fe powder and the residual solids obtained after sonication for 3, 12, and 24 h. In the supernatants of the samples sonicated for 3 and 12 h, both iron oxide nanoparticles produced by the reaction and finely fragmented iron particles were observed. After 24 h of ultrasonic irradiation, spherical particles with a mean diameter of 31 nm were obtained (Fig. S4 in the Supporting Information provides detailed particle size distributions). After 72 h, the mean particle size increased to 35 nm, and coarser particles larger than 50 nm were observed. Prolonged ultrasonic irradiation led to particle coarsening and a broader particle size distribution. In addition, many particles were observed on the surface of the residual bulk iron after long irradiation periods. These particles cover the reactive interface required for the reaction, thereby reducing the reaction rate.

Although the present route avoids iron-salt precursors, additional reagents, and counteranion-derived byproducts, the current results do not establish its overall environmental or energy advantage over conventional synthesis methods. At 43 kHz, the calorimetric acoustic power delivered to the liquid was 13.1 W, corresponding to approximately 0.31 and 0.94 kWh of acoustic energy for irradiation times of 24 and 72 h, respectively. These values represent the acoustic energy transferred to the reaction medium rather than the total electrical energy consumption of the ultrasonic system. Because the isolated yield of detached spinel-type iron oxide nanoparticles could not be quantified independently from residual Fe and surface-bound oxidation products, the energy requirement per gram of isolated nanoparticle product cannot yet be reliably determined.

Representative co-precipitation routes for spinel-type iron oxide nanoparticles employ soluble iron salts together with alkaline reagents. Saragi et al. used FeCl_2_·4H_2_O and FeCl_3_·6H_2_O with NH_3_, followed by washing with n-hexane and ethanol [15]. Patekari et al. employed FeCl_3_ and FeSO_4_ with NH_3_, followed by washing with water [19]. Nnadozie and Ajibade used FeCl_2_·4H_2_O and FeCl_3_·6H_2_O with NaOH, followed by washing with water and acetone [21]. Some conventional procedures also include high-temperature post-treatment. In contrast, the present approach uses elemental Fe powder and water without soluble Fe precursors or added precipitating agents. As summarized in Table S3, the principal process-level distinction is therefore between conventional soluble-Fe-precursor-based chemical precipitation and the present acoustic-cavitation-assisted direct metal-to-oxide transformation. This difference simplifies the chemical inputs and avoids a washing step in the present process. The relatively long ultrasonic irradiation time means that the present results do not yet demonstrate an energetic advantage over conventional co-precipitation. A complete comparison will require total electrical-energy measurements and quantitative nanoparticle productivity. Quantitative evaluation of nanoparticle recovery efficiency, electrical energy consumption per unit mass of isolated product, and approximate operating costs will also be required to assess the economic and environmental feasibility of the process. From a scale-up perspective, efficient and spatially uniform delivery of acoustic energy remains a major challenge because cavitation intensity depends strongly on reactor geometry, liquid volume, transducer configuration, and acoustic attenuation. Therefore, optimization of irradiation time, solid loading, acoustic power density, and reactor design, including continuous-flow or multi-transducer configurations, will be necessary before practical scale-up can be evaluated.

### 3.2 Effect of reaction temperature and frequency

**Fig. 3:**
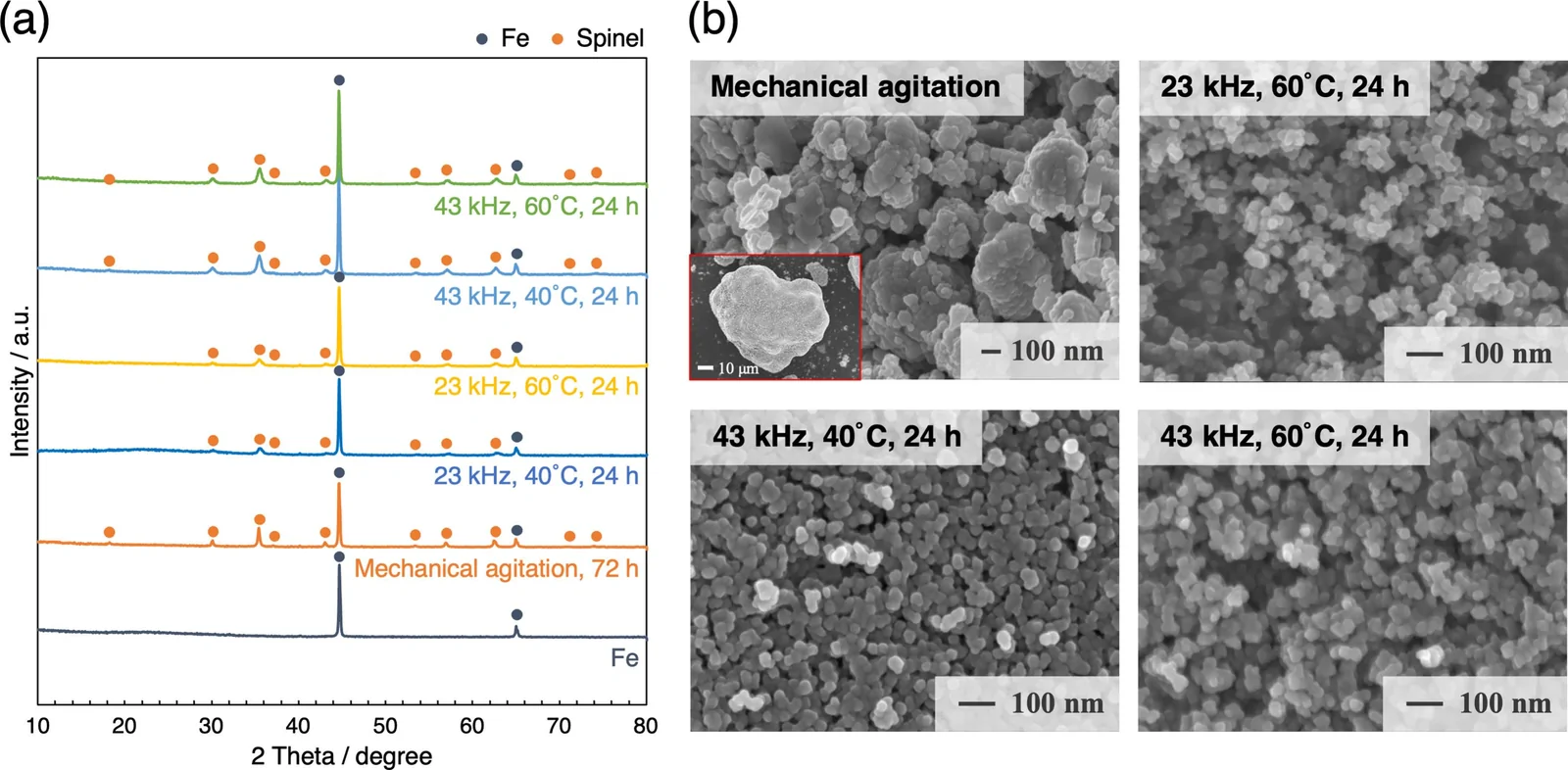
Effects of reaction temperature and ultrasonic frequency on the formation of spinel-type iron oxide from Fe powder in water: (a) XRD patterns of the overall recovered products and (b) SEM images of powders recovered from the supernatant. The results obtained by mechanical stirring are included for comparison.

Fig. 3**(a)** shows the XRD patterns of the overall products, and Fig. 3**(b)** shows SEM images of the powders recovered from the supernatant for samples synthesized at different reaction temperatures and ultrasonic frequencies. For comparison, the results obtained by mechanical stirring are also included. Spinel diffraction peaks were observed under all experimental conditions, indicating the formation of iron oxide. For the ultrasonically synthesized samples, the refined lattice parameters of the spinel phase were 8.378–8.384 Å (Table S1 and Table S2), whereas the mechanically stirred sample showed a value of 8.394 Å (Table S1). The reported lattice parameters of Fe_3_O_4_ and γ-Fe_2_O_3_ are 8.396 Å and 8.352 Å, respectively [41]. The refined values for the ultrasonically synthesized samples lie between those of Fe_3_O_4_ and γ-Fe_2_O_3_ and are slightly closer to that of Fe_3_O_4_. Because Fe_3_O_4_ and γ-Fe_2_O_3_ exhibit very similar spinel structures and diffraction patterns, particularly for nanocrystalline materials, these results do not permit an unambiguous quantitative distinction between the two phases. The refined values may be consistent with an Fe_3_O_4_-rich composition; however, this assignment remains tentative because the two spinel phases cannot be unambiguously distinguished by the present measurements. Under the present fixed nominal power condition, the 43 kHz system yielded stronger spinel-type iron oxide diffraction signals. Note that the calorimetric power was slightly higher at 43 kHz than at 23 kHz (13.1 and 12.5 W; Fig. S2). The difference in spinel diffraction intensity cannot be attributed solely to ultrasonic frequency, because differences in acoustic coupling and effective power delivered to the reaction system may also contribute to the observed behavior. The mean particle diameters were approximately 30, 32, and 33 nm at 30, 40, and 60 °C, respectively, indicating that reaction temperature had only a limited influence on the mean particle size within the investigated temperature range of 30–60 °C (Fig. S5 and Fig. S6). No substantial change in the refined spinel lattice parameter was observed with changing reaction temperature, indicating that the crystal structure of the spinel phase was relatively insensitive to temperature within the investigated range. Although the products synthesized by ultrasonic irradiation consisted of fine spherical particles, the mechanically stirred sample exhibited submicrometer-sized particles attached to the surface of bulk Fe. Accordingly, under the examined conditions, ultrasonic irradiation promoted the formation and detachment of finer particles than mechanical stirring.

The present work was designed as a proof-of-concept study to demonstrate the direct sonochemical formation of spinel-type iron oxide nanoparticles from Fe powder and water rather than as a quantitative evaluation of an optimized production process. For the temperature series conducted at 43 kHz for 24 h, Rietveld refinement indicated estimated Fe-to-spinel conversions of 36.1, 68.5, and 63.7% at 30, 40, and 60 °C, respectively, assuming Fe_3_O_4_ stoichiometry for the spinel phase. Thus, although the mean particle size and refined spinel lattice parameter changed only slightly over the investigated temperature range, the extent of Fe conversion was substantially lower at 30 °C. This result indicates that reaction temperature affects the extent of Fe oxidation and spinel formation more strongly than the mean particle size under the present conditions. However, these conversion values represent conversion to the spinel oxide phase and not the isolated yield of detached nanoparticles. Because residual metallic Fe, surface-bound oxidation products, detached spinel nanoparticles, and potentially fragmented Fe particles coexist in the recovered solids, quantitative determination of the isolated nanoparticle yield requires further phase- and morphology-resolved mass-balance analysis. Measurements of reaction kinetics, dissolved Fe species, and radical formation will also be required to further evaluate the reaction mechanism and process efficiency.

### 3.3 Magnetic properties

**Fig. 4:**
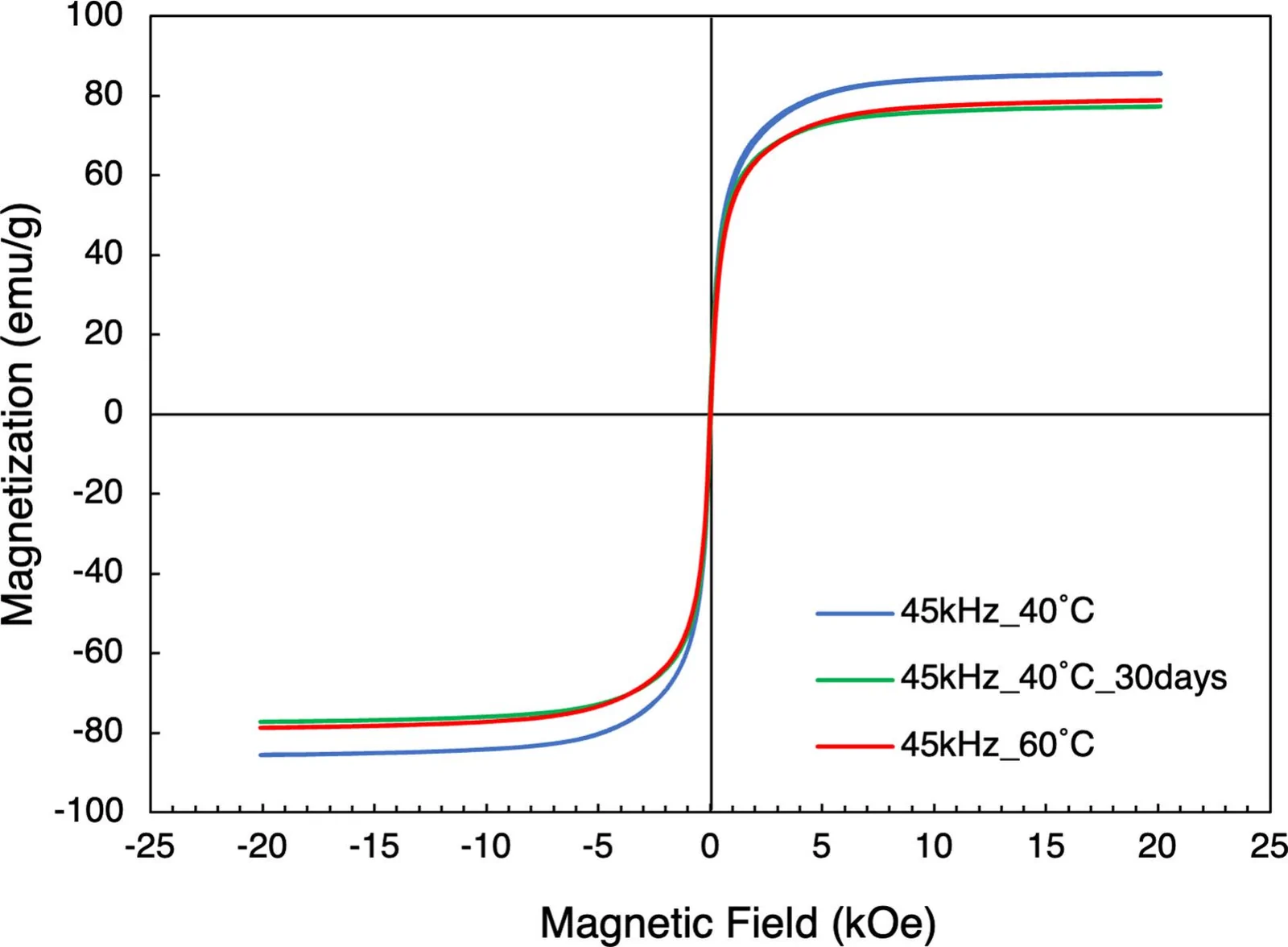
Magnetization curves measured at 300 K for spinel-type iron oxide products synthesized by ultrasonic irradiation of Fe powder in water at 43 kHz and 40 or 60 °C. The magnetization curve of the sample synthesized at 40 °C and measured after 30 days of storage is also shown.

Magnetic measurements were performed on samples synthesized at 43 kHz, which exhibited stronger spinel-type iron oxide diffraction signals. Fig. 4 shows the magnetization curves of the iron oxide samples measured at 300 K using a SQUID. The saturation magnetization (*M*_s_) measured at the maximum applied magnetic field was 85.6 emu/g for the sample synthesized at 40 °C and 76.9 emu/g for the sample synthesized at 60 °C. The negligible coercivity and remanent magnetization observed for these fine particles are consistent with a superparamagnetic-like magnetic response at room temperature. The magnetic behavior of iron oxide nanoparticles is influenced not only by particle size but also by phase composition, interparticle interactions, surface structure, and structural defects. The literature values of saturation magnetization for Fe_3_O_4_ and γ-Fe_2_O_3_ are reported to be 92 emu/g and 74–80 emu/g [2]. The magnetization of 85.6 emu/g obtained for the sample synthesized at 40 °C was lower than that of bulk Fe_3_O_4_ but higher than the typical range for γ-Fe_2_O_3_. This intermediate value may reflect the coexistence of Fe_3_O_4_ and γ-Fe_2_O_3_. Finite-size effects, spin canting or disorder at the nanoparticle surface, and cavitation- or oxidation-induced defects may also reduce the magnetization relative to bulk Fe_3_O_4_. Because the surface spin structure and defect concentration were not directly characterized, their individual contributions cannot be distinguished in the present study.

The decrease in saturation magnetization from 85.6 to 75.6 emu/g after 30 days of storage may be consistent with further oxidation of regions tentatively assigned as Fe_3_O_4_-rich during exposure to air. Because only the initial and 30-day measurements were obtained, the kinetics and extent of this transformation cannot be determined from the present data. Longer-term magnetic measurements with intermediate sampling points will be required to quantitatively evaluate the storage stability and oxidation behavior of the nanoparticles. Definitive quantitative identification of the Fe_3_O_4_/γ-Fe_2_O_3_ ratio would require additional valence-sensitive characterization, such as Mössbauer spectroscopy or X-ray photoelectron spectroscopy.

### 3.4 Proposed formation pathway of spinel-type iron oxide nanoparticles

**Fig. 5:**
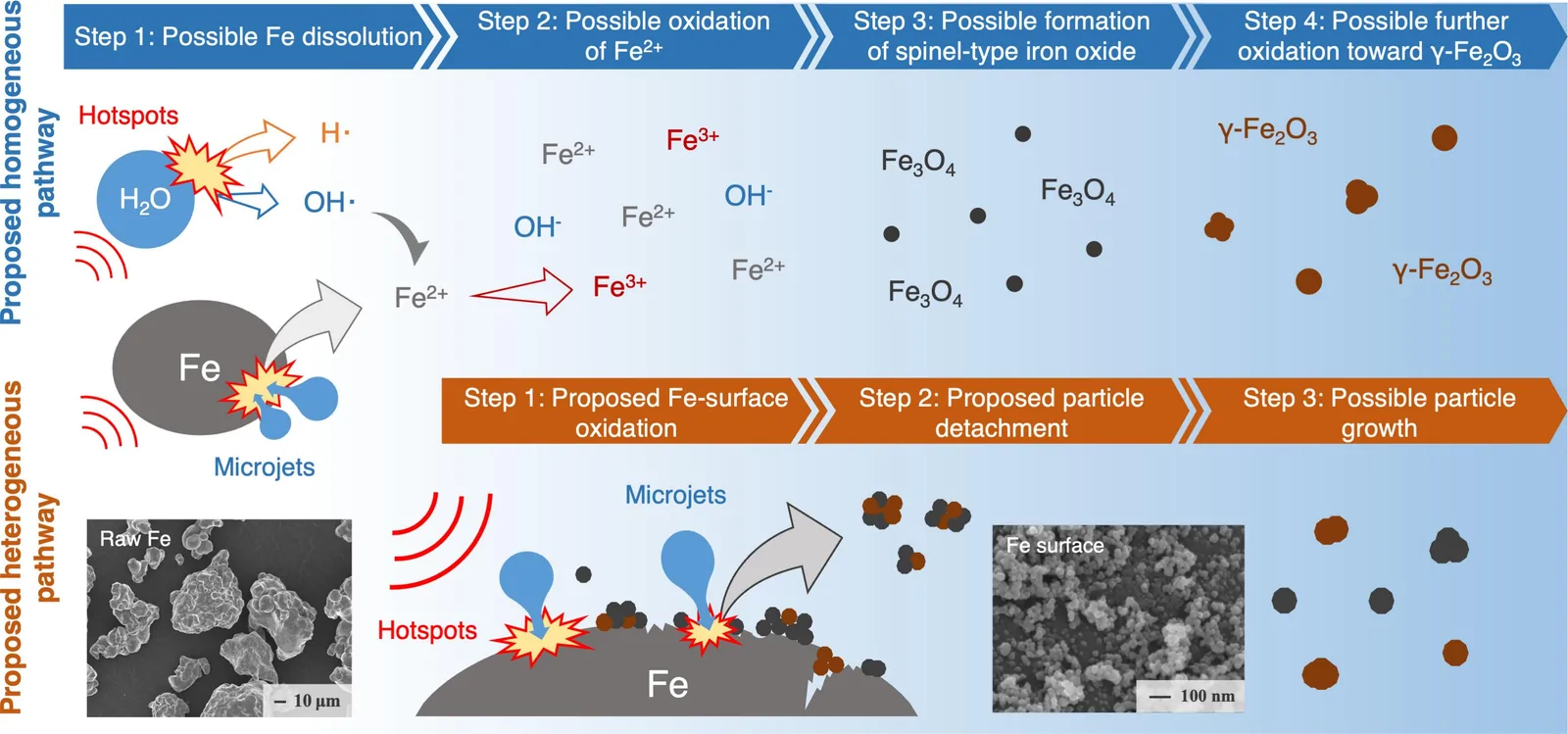
Schematic illustration of the proposed formation pathway of spinel-type iron oxide nanoparticles from Fe powder in water under ultrasonic irradiation. The scheme illustrates plausible homogeneous and heterogeneous pathways involving cavitation-assisted surface renewal, Fe dissolution and oxidation, and particle formation and detachment. The involvement of radical species, H_2_O_2_, and dissolved Fe^2+^/Fe^3+^ intermediates in the proposed pathway is hypothetical because these species were not directly quantified in the present study.

Our scientific hypothesis for the formation pathway is that the reaction scheme proposed in Fig. 5 represents a plausible sequence of processes consistent with the experimentally observed particle morphology and phase evolution, together with previously reported sonochemical reactions in water. Radical species, H_2_O_2_, and dissolved Fe^2+^/Fe^3+^ intermediates were not directly quantified in the present study; therefore, the individual elementary reactions shown in Eqs. (1), (2), (3), (4), (5), (6), (7), (8) should be regarded as hypothetical reaction steps rather than experimentally established reactions. In the present system, both homogeneous and heterogeneous nucleation may contribute to particle formation. In the proposed homogeneous nucleation pathway, iron species may be released from Fe particles and dissolve into the aqueous phase as ions or clusters. Solid Fe may undergo oxidation and release Fe^2+^ species into the aqueous phase. In addition to the chemical effects of acoustic cavitation, the intense mechanical effects associated with microjets and shock waves may promote fragmentation, erosion, and renewal of the Fe surface. Similar mechanically induced particle refinement and phase transformation have been reported for iron oxides subjected to high-energy ball milling [18].

(1) $$2Fe_{\left(solid\right)}+O_{2}+4H^{+}\to 2Fe^{2+}+2H_{2}O$$

The physical effects of ultrasound may also promote the detachment of Fe particles and clusters, thereby increasing the available reaction interface area. In addition, ultrasonic irradiation may promote water sonolysis within cavitation hotspots, generating hydroxyl radicals that can recombine to form hydrogen peroxide, as reported in aqueous sonochemical systems [26].

(2) $$H_{2}O\to H∙+OH∙$$

(3) $$2OH∙\;\to H_{2}O_{2}$$

The hydroxyl radicals and hydrogen peroxide may act as oxidizing species, facilitating the conversion of Fe^2+^ into Fe^3+^. The recombination of hydroxyl radicals can produce hydrogen peroxide, which has been reported to oxidize Fe^2+^ to Fe^3+^ during sonoelectrochemical synthesis of magnetite [42].

(4) $$Fe^{2+}+OH∙\;\to Fe^{3+}+OH^{-}$$

(5) $$2Fe^{2+}+\;H_{2}O_{2}\to 2Fe^{3+}+2OH^{-}$$

In the proposed pathway, reactions involving Fe^2+^ and Fe^3+^ species may lead to the formation of Fe_3_O_4_ crystal nuclei.

(6) $$Fe^{2+}+2Fe^{3+}+8OH^{-}\to Fe_{3}O_{4}+4H_{2}O$$

Changes in reaction temperature may alter cavitation behavior and the formation of reactive species. Such reactive species may contribute to the oxidation of Fe^2+^ to Fe^3+^. The oxidation of Fe_3_O_4_ to γ-Fe_2_O_3_ has been reported to proceed via solid-state diffusion of ions [43], and prolonged reaction may promote further oxidation of Fe_3_O_4_-rich regions.

(7) $$4Fe_{3}O_{4}+O_{2}\to 6\gamma Fe_{2}O_{3}$$

In the proposed heterogeneous pathway, reactions may occur primarily on the surface of the Fe particles. As Fe has a high tendency to ionize, it is easily corroded in water [44], resulting in the formation of an oxide layer [45].

(8) $$3Fe+4H_{2}O\to Fe_{3}O_{4}+4H_{2}$$

The fine particles observed on the bulk Fe surface in Fig. 2**(b)** are consistent with corrosion-derived oxidation products associated with the surface oxide layer. The physical effects of ultrasonic irradiation may disrupt this oxide layer and expose fresh Fe surfaces [39]. Such newly exposed Fe surfaces may then provide additional reaction interfaces with water, promoting further formation of iron oxide. In the case of mechanical stirring, Fe dissolution may have been more limited, and oxidation appears to have proceeded predominantly at the bulk Fe surface. The limited detachment of the surface oxide layer may have reduced the formation of newly exposed reaction interfaces. Under these conditions, continued oxidation and growth of surface-bound products may have contributed to the formation of the submicrometer-sized particles observed after mechanical stirring.

## 4 Conclusions

In this study, a sonochemical process for synthesizing iron oxide nanoparticles from coarse Fe powder under relatively mild aqueous conditions is reported. This study aimed to demonstrate a simple sonochemical route for the direct formation of spinel-type iron oxide nanoparticles from metallic Fe and water without auxiliary chemical reagents and to clarify the role of the ultrasonic reaction field in this process. Fine iron oxide nanoparticles were successfully produced by ultrasonic irradiation of Fe powder in water. The magnetization of the product synthesized at 40 °C was 85.6 emu/g, whereas that synthesized at 60 °C was 76.9 emu/g. This difference may reflect changes in the Fe^2+^/Fe^3+^ balance and/or nanoscale magnetic effects; however, the phase composition could not be determined quantitatively from the present measurements. The decrease in magnetization after 30 days of storage may be consistent with further oxidation of regions tentatively assigned as Fe_3_O_4_-rich during exposure to air, although the phase evolution and oxidation kinetics could not be determined quantitatively from the present measurements. The sonochemical process proposed in this study uses metallic Fe as the starting material and water as the reaction medium without requiring additional chemical reagents. This approach simplifies the reaction system and potentially reduces reagent-derived wastewater associated with conventional salt-derived precursor routes. The scientific contribution of this work lies in demonstrating an acoustic-cavitation-assisted direct metal-to-oxide transformation of elemental Fe into nanoscale spinel-type iron oxide particles, distinct from conventional soluble-Fe-salt-based precipitation routes. Rietveld refinement indicated substantial conversion of Fe to the spinel oxide phase under the investigated conditions. However, the present study represents a proof of concept and does not establish an overall energetic, environmental, or economic advantage over conventional synthesis routes. Further quantitative studies of isolated nanoparticle yield, mass balance, total electrical energy consumption, productivity, reaction kinetics, dissolved Fe species, and radical formation will be required to evaluate process efficiency and scalability and to further validate the proposed formation pathway.

## CRediT authorship contribution statement

**Madoka Yoshikawa:** Writing – original draft, Visualization, Methodology, Investigation, Formal analysis, Data curation. **Hirotsugu Takizawa:** Supervision. **Yamato Hayashi:** Writing – review & editing, Validation, Supervision, Project administration, Methodology, Formal analysis, Data curation, Conceptualization.

## Declaration of competing interest

The authors declare that they have no known competing financial interests or personal relationships that could have appeared to influence the work reported in this paper.
