# Supplementary material for: Reagent-free sonochemical synthesis of iron oxide nanoparticles from iron powder and water promoted by acoustic cavitation
Source: Ultrason Sonochem. 2026 Aug 25;133:108028. doi: 10.1016/j.ultsonch.2026.108028 (PMC13546830; doi:10.1016/j.ultsonch.2026.108028)
Supplement: Supplementary file 1 [file mmc1.docx]

**Reagent-Free Sonochemical Synthesis of Iron Oxide Nanoparticles from Iron Powder and Water Promoted by Acoustic Cavitation**

*Madoka Yoshikawa, Hirotsugu Takizawa, Yamato Hayashi*^*^

*Graduate School of Engineering, Department of Applied Chemistry, Tohoku University, 6-6 Aoba, Aramaki, Aobaku, Sendai 980-8579, Japan*

^*^ Corresponding author: Y. Hayashi; E-mail address: [yamato.hayashi.b6@tohoku.ac.jp](mailto:yamato.hayashi.b6@tohoku.ac.jp)

**Fig. S1** The schematic of the ultrasound reaction system. The water bath temperature was kept constant using a circulator. Ultrasound was applied indirectly from the bottom of the flask.

**Fig. S2** The water temperature inside the flask under ultrasonication at (a) 23 kHz, (b) 43 kHz with 150 W power. The nominal power was defined by RF power amplifier. The rate of temperature rise in the flask was determined from the slope of a linear fit, and the calorimetric power was calculated using the following equation:

$$P=c_{P}\times m\times\left( \frac{dT}{dt} \right)$$

where *P*, *c_p_*, *m*, *T* and *t* represent the calorimetric power, heat capacity of water (4.18 J/g °C at 30 °C), mass of water, temperature, and irradiation time, respectively. The calculated calorimetric power was 12.5 W at 23 kHz and 13.1 W at 43 kHz.

**Fig. S3** XRD pattern fitting via Rietveld refinement using a modified split pseudo-Voigt function.

**Fig. S4**. Particle-size distributions of the products obtained after different ultrasonic irradiation times at 23 kHz and 40 °C. Particle sizes were determined from SEM images using ImageJ based on the equivalent circular diameter calculated from the projected particle area. The mean particle sizes were 31 ± 4.7 nm (n = 732) after 24 h and 35 ± 7.4 nm (n = 642) after 72 h, where the values are expressed as mean ± standard deviation (SD) and n represents the number of particles analyzed.

**Fig. S5**. Particle-size distributions of the products synthesized at different reaction temperatures at 43 kHz for 24 h. Particle sizes were determined from SEM images using ImageJ based on the equivalent circular diameter calculated from the projected particle area. The mean particle sizes were 32 ± 4.7 nm (n = 677) at 40 °C and 33 ± 7.4 nm (n = 515) at 60 °C, where the values are expressed as mean ± standard deviation (SD) and *n* represents the number of particles analyzed.

**Fig. S6**. SEM image, particle-size distribution, and Rietveld refinement of the XRD pattern for the sample synthesized by ultrasonic irradiation at 43 kHz and 30 °C for 24 h. Particle sizes were determined from SEM images using ImageJ based on the equivalent circular diameter calculated from the projected particle area. The mean particle size was approximately 30 ± 3.6 nm (n = 608), where the value is expressed as mean ± standard deviation (SD) and n represents the number of particles analyzed.

**Table S1.** Refined mass fractions and lattice parameters of metallic Fe and the spinel-type iron oxide obtained by Rietveld refinement, together with the Fe conversion estimated assuming Fe_3_O_4_ stoichiometry for the spinel phase.

| Frequency | 23 kHz | | | 43 kHz | | Mechanical agitation |
| --- | --- | --- | --- | --- | --- | --- |
| Temperature | 40 °C | | 60 °C | 40 °C | 60 °C | 40 °C |
| Time | 24 h | 72 h | 24 h | 24 h | | 72 h |
| Mass fractions (Fe) | 0.1725 | 0.0616 | 0.2854 | 0.2501 | 0.2922 | 0.5077 |
| Mass fractions (spinel) | 0.8275 | 0.9384 | 0.7146 | 0.7499 | 0.7078 | 0.4923 |
| Lattice parameter of spinel (Å) | 8.381 | 8.383 | 8.382 | 8.378 | 8.379 | 8.394 |
| % Fe converted | 77.6 | 91.7 | 64.4 | 68.5 | 63.7 | 41.2 |

**Table S2.** Phase fractions, lattice parameter of the spinel phase, and estimated Fe conversion obtained from Rietveld refinement of the XRD pattern for the sample synthesized by ultrasonic irradiation at 43 kHz and 30 °C for 24 h.

| Mass fractions (Fe) | Mass fractions (spinel) | Lattice parameter of spinel (Å) | % Fe converted |
| --- | --- | --- | --- |
| 0.5612 | 0.4388 | 8.384 | 36.1 |

**Table S3.** Comparison of representative conventional iron-salt-based precipitation routes and the present reagent-free sonochemical process.

|  | Conventional iron-salt-based process | **Present process** |
| --- | --- | --- |
| **Fe source** | FeCl_2_, FeCl_3_, FeSO_4_, etc. | **Fe powder** |
| **Reaction medium** | Aqueous solution | **Water** |
| **Precipitating / pH-adjusting agent** | NH_3_, NaOH, etc. | **Not required** |
| **Additional chemical reagent for nanoparticle formation** | Generally required depending on the method | **Not required** |
| **Driving process** | Chemical precipitation | **Acoustic cavitation-assisted solid–liquid reaction** |
| **Reaction temperature** | Ambient–100 °C | **40 °C, atmospheric pressure** |
| **Representative reaction time** | Method-dependent | **24 h** |
| **Washing process** | Generally required | **No washing step used in the present process** |
| **Main distinguishing feature** | Soluble Fe precursor → precipitation | **Elemental Fe → spinel-type iron oxide directly in water** |
